# NMR Study of the O-Specific Polysaccharide and the Core Oligosaccharide from the Lipopolysaccharide Produced by *Plesiomonas shigelloides* O24:H8 (Strain CNCTC 92/89)

**DOI:** 10.3390/molecules20045729

**Published:** 2015-04-01

**Authors:** Lena C. E. Lundqvist, Marta Kaszowska, Corine Sandström

**Affiliations:** 1 Department of Chemistry and Biotechnology, Uppsala BioCenter Swedish University of Agricultural Sciences, P.O. Box 7015, Uppsala SE-750-07, Sweden; E-Mail: Lena.Lundqvist@slu.se; 2 Ludwik Hirszfeld Institute of Immunology and Experimental Therapy, Polish Academy of Sciences, R. Weigla 12, Wroclaw PL-53-114, Poland; E-Mail: marta.kaszowska@iitd.pan.wroc.pl

**Keywords:** lipopolysaccharide, *Plesiomonas shigelloides*, NMR, ESI MS, structural analysis

## Abstract

The structures of the O-specific polysacccharide and core oligosaccharide of the lipopolysaccharide from *Plesiomonas shigelloides* O24:H8, strain CNCTC 92/89, have been investigated by NMR spectroscopy and ESI mass spectrometry. The O-specific polysaccharide was found to be composed of a tetrasaccharide repeating unit consisting of [→3)-α-Fuc*p*NAc-(1→3)-α-Gal*p*NAcA-(1→3)-α-Qui*p*NAc-(1→] and of α-Rha*p*NAc (1→4) linked to the Gal*p*NAcA residue. An identical structure has been reported for the capsular polysaccharide of the clinical isolate of *Vibrio vulnificus* strain BO62316 [1]. The core oligosaccharide was composed of a decasaccharide which structure is identical with these in *P. shigelloides* serotype O54 [2] and serotype O37 [3].

## 1. Introduction

*Plesiomonas shigelloides* is a Gram-negative bacterium, flagellated and with a relatively straight rod-shape, that is widely distributed in nature. *P. shigelloides* was formerly classified in the *Vibrionaceae* family, but it now belongs to the *Enterobacteriaceae* family [4]. This bacterium is found in fresh water systems mainly in tropical and subtropical climates [5], but has also been found in fresh water samples in northern Europe [6]. The bacteria occur as free-living cells in water or in water-dwelling organisms such as fish, crabs, prawns, mussels and oysters. *P. shigelloides* is an opportunistic pathogen and causes gastrointestinal illness. It is one of the most frequent causes of traveler’s diarrhea in Japan and China [5]. Humans are mostly infected by *P. shigelloides* through drinking untreated water or eating uncooked shellfish [7]. The pathogenicity of *P. shigelloides* is not completely understood. There are several reported possible virulence factors; the cholera-like toxin [8], thermo stable and thermo labile toxins [9], β-haemolysin [10] and a cytotoxic protein [11], where the lipopolysaccharide (LPS) is one of the main virulent factors.

The LPS is the major component of the outer cell membrane of the Gram-negative bacteria and is essential to the function. The LPS can be divided into three separate regions that differ in structure and function: lipid A, core oligosaccharide and O-specific chain. These three regions are all significant for the biological activity and involvement in host-bacterium interactions. The biological activity of the LPS depends mainly on the chemical structure of lipid A and is only modulated by the polysaccharide part. The core oligosaccharide (OS) is important for biological and physical properties of the overall LPS and plays a significant role in interactions with the host. The O-specific polysaccharide (O-specific chain, O-antigen) determines the bacterial O-serotype, and defines a fingerprint of the bacteria [12]. Because of the LPS role in the pathogenesis mechanism, it is of value to expand the knowledge about the structure of the LPS from *P. shigelloides* [13] Here we present the structural study of the repeating unit and of the core oligosaccharide of the *P. shigelloides* O24 LPS.

## 2. Results and Discussion

**Figure 1:**
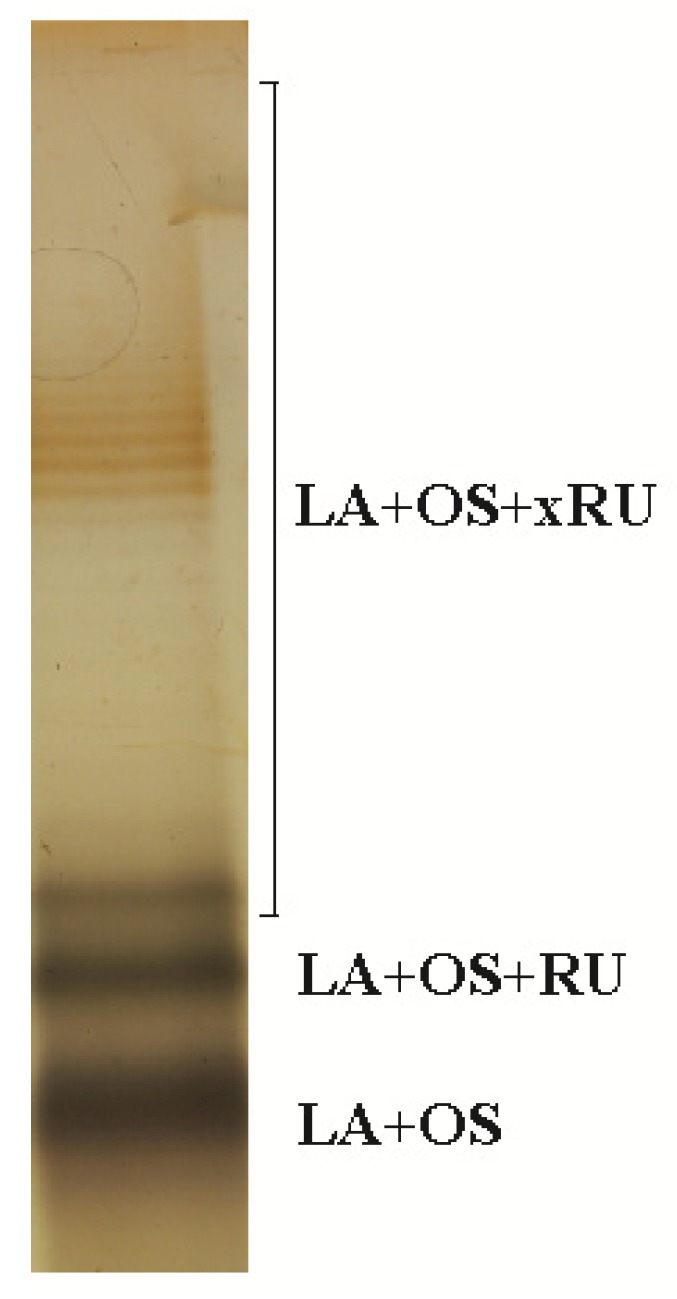
SDS/PAGE analysis of the lipopolysaccharide isolated from *P. shigelloides* O24 (CNCTC 92/89). The bands were visualized by silver-staining. LA-lipid A, OS-core oligosaccharide, RU-repeating unit.

The LPS of *P. shigelloides* O24 (CNCTC 92/89), isolated by hot phenol/water extraction, was shown to have a smooth character using SDS-PAGE. The different bands observed on the silver stained gel correspond to components in LPS consisting of unsubstituted core oligosaccharide (LA+OS) as well as core oligosaccharide substituted with different number of repeating units (LA+xRU) (Figure 1).

### 2.1. Isolation of the O-Specific Polysaccharide

The PS was liberated through mild acidic hydrolysis and isolated by gel filtration on a Superdex 75 column. Three main fractions were obtained and further separated on a Superdex 30 column to yield fraction of PS (yield 7.4% of LPS), OSI (yield 4.6%), and OSII (yield 19.6%). The fractions were analyzed by NMR spectroscopy and ESI-MS which showed that the PS fraction consisted of the core oligosaccharide substituted with different numbers of repeating units (RU) while the OSI fraction consisted of the core oligosaccharide with one repeating unit and the OSII fraction of the unsubstituted core oligosaccharide.

### 2.2. Structure Analysis of the O-Specific Polysaccharide

**Figure 2:**
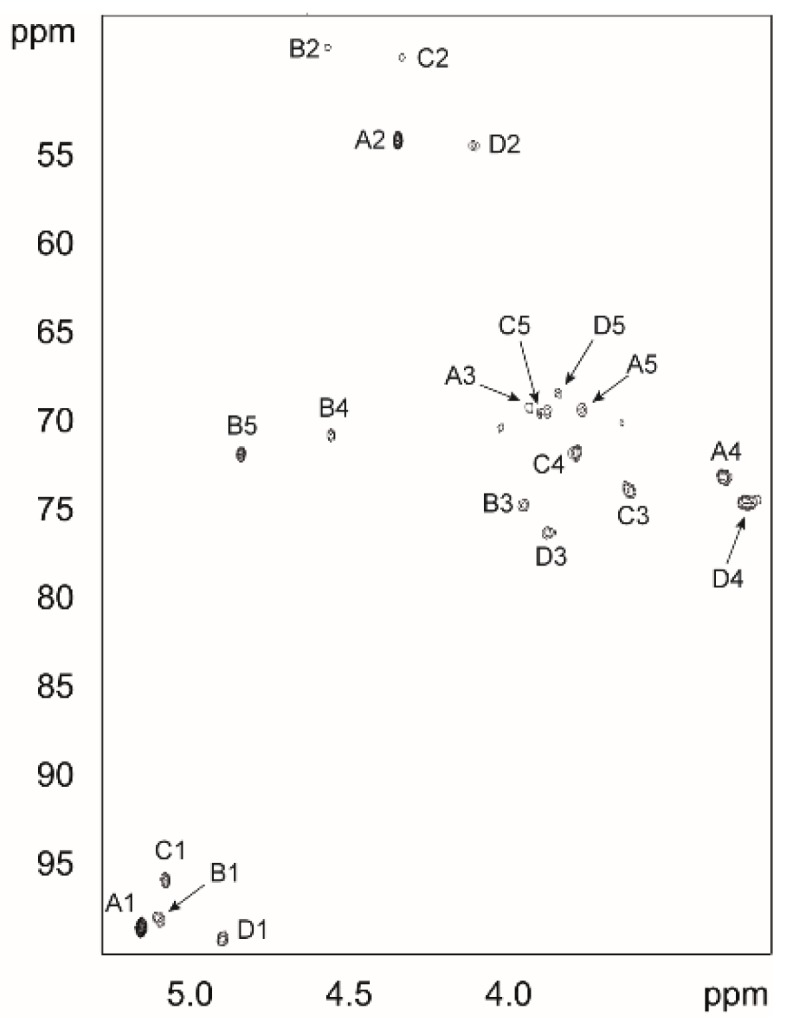
Part of the HSQC spectrum of the O-specific polysaccharide of the LPS from *P. shigelloides* O24. The inset shows the anomeric region of the spectrum. The uppercase letters refer to designation of sugar residues.

The ^1^H-NMR spectra of PS showed four major signals in the region for anomeric protons (5.4–4.8 ppm) indicating tetrasaccharide repeating units. The four sugar residues are designated **A**–**D** according to decreasing proton chemical shift values. The ^1^H, ^13^C-HSQC spectrum of the PS showed four cross-peaks in the region for anomeric resonances (Figure 2) at δ_H_/δ_C_ 5.18/98.5 ppm (residue **A**), δ_H_/δ_C_ 5.13/97.9 ppm (residue **B**), δ_H_/δ_C_ 5.11/95.7 ppm (residue **C**) and δ_H_/δ_C_ 4.93/99.1 ppm (residue **D**), corresponding to hexapyranosyl residues and confirming that the polysaccharide consisted of tetrasaccharide repeating units. The ^1^H and ^13^C resonances of the four constituent sugar residues (Table 1) were assigned using a combination of 2D NMR experiments as well as by comparison with previously published ^1^H- and ^13^C-NMR data for mono- and polysaccharides. The HSQC spectrum contained signals for three methyl groups at δ_H_ 1.22–1.26 ppm and δ_C_ 16.4–17.6 ppm corresponding to H6 and C6 of 6-deoxyhexoses as well as four signals at δ_H_ 1.87–2.05 ppm and δ_C_ 23.1–23.6 ppm characteristic of methyl groups from the acetyl group in acetamido sugars.

The four sugars had small ^3^*J*_1,2_ coupling constant values indicating that they are α-linked. The ^1^*J*_C1, H1_ values, larger than 170 Hz and obtained from a HSQC experiment without carbon decoupling, confirmed the α-pyranosyl configuration for all four residues; **A** (~175 Hz), **B** (~179 Hz), **C** (~174 Hz), and **D** (~172 Hz). In the TOCSY spectra, residue **A** showed correlations between H1 and H2 and from H6 to H2. Residue **A** had small ^3^*J*_1,2_ and ^3^*J*_2,3_ but large ^3^*J*_3,4_ and ^3^*J*_4,5_ indicating a *manno*-configuration. The α-configuration was confirmed by the observation in the NOESY spectra of H1-H2 and H1-CH_3_ cross-peaks but not of H1-H3 or H1-H5 cross-peaks. For residues **B** and **C**, correlations from H1 to H4 were observed in the TOCSY spectra. Residue **B** was assigned to have the *galacto*-configuration and the downfield shifts of the H4 and H5 at δ_H_ 4.59 and 4.87 ppm were characteristics for galacturonic acid residue. Residue **C** had the *galacto*-configuration and this together with the ^1^H and ^13^C chemical shifts of the methyl group at δ_H_/δ_C_ 1.22/16.4 ppm gave the *N*-acetyl fucosamine residue. In the TOCSY spectra, cross-peak between H6 and H5 of **C** was observed. The H1 to H6 correlation in the TOCSY spectra together with the ^1^H and ^13^C chemical shifts indicated that residue **D** is a 2-acetamido-2,6,-dideoxy-α- glucopyranose or α-Qui*p*NAc. The positions of the amino groups in the four sugar residues were confirmed by correlations in the HSQC spectra of the H2 protons at the nitrogen-bearing carbons to the corresponding C2 carbons at δ_C_ 48.8–54.4 ppm.

**Table 1:** ^1^H- and ^13^C-NMR chemical shifts (ppm) of the PS fraction from *P. shigelloides* O24. Spectra were obtained for D_2_O solutions at 35 °C.

Residue		Chemical Shifts (ppm) for						
		H1	H2	H3	H4	H5	H6	NAc
		C1	C2	C3	C4	C5	C6	
**A**	α-Rha*p*NAc-(1→	5.18	4.37	3.97	3.36	3.73	1.23	2.05 *
		98.5	54.1	69.0	72.9	69.7	17.6	175.1 *
**B**	→3,4)-α-Gal*p*NAcA-(1→	5.13	4.60	3.98	4.59	4.87	----	2.08
		97.9	48.8	74.7	70.6	71.7	----	174.9
**C**	→3)-α-Fuc*p*NAc-(1→	5.11	4.37	3.65	3.82	3.92	1.22	2.05 *
		95.7	49.4	73.7	71.7	67.4	16.4	175.1 *
**D**	→3)-α-Qui*p*NAc-(1→	4.93	4.14	3.91	3.29	3.79	1.26	1.99
		99.1	54.4	76.2	74.5	69.4	17.6	173.9

*: Could not be differentiated.

The sequence of the sugar residues and the connections between them within the repeating unit of the O-polysaccharide was obtained by assignment of the inter-residue interactions observed in the ^1^H, ^1^H-NOESY and ^1^H, ^13^C-HMBC spectra. Inter-residues NOEs were found between H1 of **A** and H4 of **B**, H1 of **B** and H3 of **D**, H1 of **D** and H3 of **C**, H1 of **C** and H3 of **B** (Table 2).

**Table 2:** Selected inter-residue NOE and ^3^*J*_H,C_-connectivities from the anomeric atoms of the PS of *P. shigelloides* O24 LPS.

Residue	H1/C1 δ_H_/δ_C_ ppm	Connectivities to		Inter-Residue Atom/Residues
		δ_H_	δ_C_	
**A** α-Rha*p*NAc-(1→	5.18	4.59	70.6	H4, C4 of **B**
**B** →3,4)-α-Gal*p*NAcA-(1→	5.13	3.91	76.2	H3, C3 of **D**
**C** →3)-α-Fuc*p*NAc-(1→	5.11	3.98	74.7	H3, C3 of **B**
**D** →3)-α-Qui*p*NAc-(1→	4.93	3.65	73.7	H3, C3 of **C**

**Figure 3:**
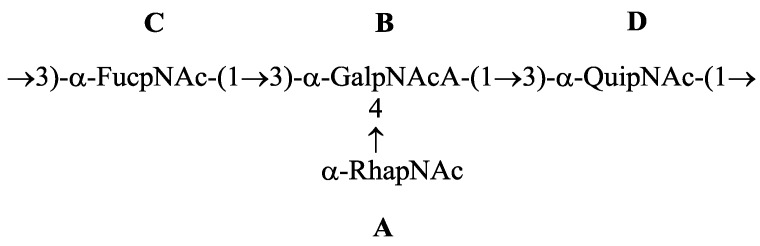
Structure of the repeating unit from P. shigelloides O24 (strain CNCTC 92/89).

This sequence was confirmed by the HMBC spectra that showed cross-peaks between the anomeric protons and the carbons at the linkage position. Based on these data, it was concluded that the tetrasaccharide repeating unit of the O-polysaccharide of *P. shigelloides* O24 had the following structure (Figure 3).

The ^1^H- and ^13^C-NMR data of the O-specific repeating unit from *P. shigelloides* O24 were identical to those obtained for the capsular polysaccharide from clinical isolate *Vibrio vulnificus* strain BO62316 [1,14] indicating that these two compounds have the same structure. The α-Fuc*p*NAc and α-Gal*p*NAcA residues were reported to have the D configuration while α-Qui*p*NAc and α-Rha*p*NAc had the l-configuration.

**Figure 4:**
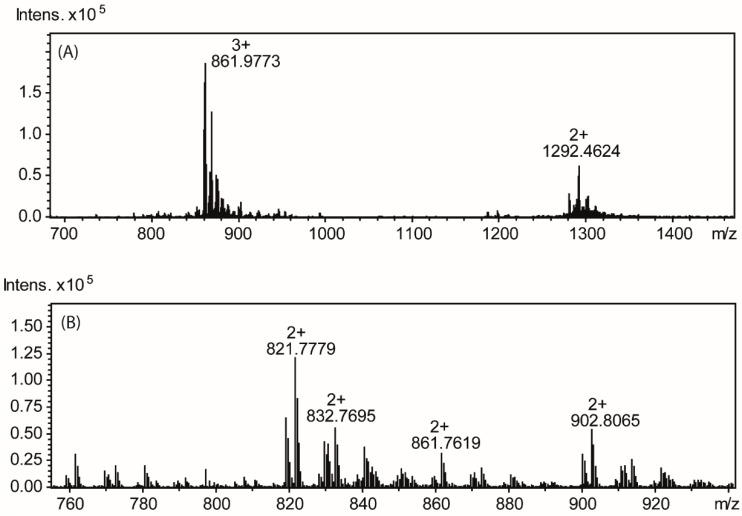
Mass spectra of (**A**) the OSI fraction containing the core oligosaccharide substituted by one repeating unit (**B**) the OSII fraction containing the unsubstituted core oligosaccharide.

The mass of the repeating unit was determined from ESI-MS by comparing the mass of the OSI fraction containing core oligosaccharide substituted by one repeating unit with the mass of the OSII fraction containing the unsubstituted core oligosaccharide. The OSI fraction showed two main ions at *m/z* 1292.46 [M-H_2_O+H+Na]^2+^ and at *m/z* 861.97 [M-H_2_O+2H+Na]^3+^ corresponding to the core oligosaccharide substituted by one repeating unit with the mass of *m/z* 2759 (Figure 4A). The OSII fraction showed an ion at *m/z* 902.81 [M-H_2_O+H+Na]^2+^, giving the monoisotopic mass of 1799.6 Da (Figure 4). These data showed that the mass of the O-specific repeating unit is 779.3 Da in good agreement with the mass expected for the structure determined from the NMR data.

### 2.3. Structure Analysis of the Core Oligosaccharide

A serological screening of 69 different O-serotypes of *P. shigelloides* reported by Niedziela *et al.* [2] suggested that epitopes similar to the core oligosaccharide of the serotype O54 (strain CNCTC 113/92) could also be present in the core oligosaccharide of the serotype O24 (strain CNCTC 92/89). Thus comparison of the MS and NMR data with those of the core oligosaccharide of *P. shigelloides* CNCTC 113/92 was done.

The ESI-MS analysis of the core oligosaccharide (OSII) showed two main ions at *m/z* 821.78 [M-H_2_O+H+Na]^2+^ and *m/z* 902.8 [M-H_2_O+H+Na]^2+^, giving the monoisotopic masses of 1637.6 and 1799.6 Da respectively, thus differing only in one hexose unit (162 Da difference) and suggesting a decasaccharide and nonasaccharide structure, respectively (Figure 4B). The nine sugar residues present in *P. shigelloides* CNCTC 113/92, two Gal, one Glc, three Hep, one GalA, one GlcN and one Kdo, give together a monoisotopic mass of 1637.52 Da, which is in agreement with the mass of OSII.

**Figure 5:**
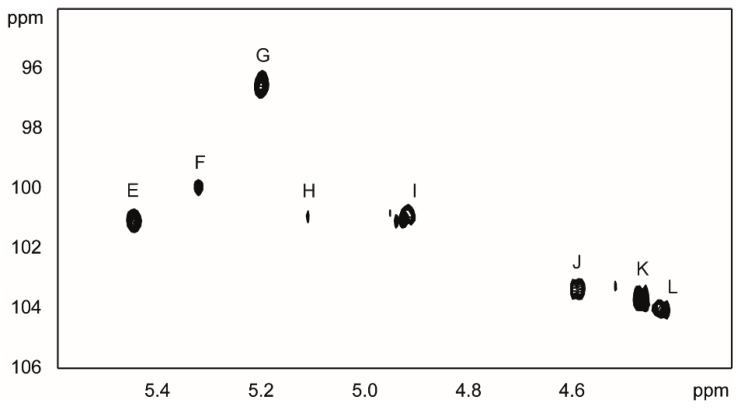
Portion of the HSQC spectrum showing the anomeric ^1^H- and ^13^C-NMR signals from the core oligosaccharide of *P. shigelloides* O24. The uppercase letters refer to designation of sugar residues.

The ^1^H-NMR and ^1^H, ^13^C-HSQC (Figure 5) spectra of the OSII fraction contained eight main signals in the region for anomeric protons and carbons as well as signals characteristic for the deoxy protons of a Kdo residue, indicating a nonasaccharide core structure. The sugar residues are designated as **E**–**M** according to decreasing proton chemical shift values as shown in Figure 4 with δ_H_/δ_C_ 5.45/101.1 ppm (residue **E**), δ_H_/δ_C_ 5.32/99.9 ppm (residue **F**), δ_H_/δ_C_ 5.21/96.50 ppm (residue **G**), δ_H_/δ_C_ 5.12/100.9 ppm (residue **H**), δ_H_/δ_C_ 4.93/100.9 ppm (residue **I**), δ_H_/δ_C_ 4.59/103.4 ppm (residue **J**), δ_H_/δ_C_ 4.47/103.7 ppm (residue **K**), δ_H_/δ_C_ 4.43/104.1 ppm (residue **L**). The major ^1^H- and ^13^C-NMR signals corresponding to the nine sugars were assigned using COSY, TOCSY, NOESY, HSQC-TOCSY and HMBC experiments. The ^1^*J*_C-1, H-1_ values confirmed the α-pyranosyl configuration for residues **E** (175 Hz), **F** (180 Hz), **G** (170 Hz) and **I** (173 Hz) and β-pyranosyl configuration for residues **J** (161 Hz) and **K** (160 Hz), and **L**. NMR analysis of the OSI fraction showed when compared to fraction OSII the presence of an additional signal in the anomeric region at δ_H_/δ_C_ 4.47/103.6 ppm and corresponding to a sugar with the glucose configuration (denoted **N**). The NOESY spectrum showed a cross-peak between H1 **N** at 4.47 ppm and H6 **G** at 4.16 ppm while HMBC showed a cross-peak between H1 **N** at 4.47 ppm and H6 **G** at 67.9 ppm demonstrating a β(1→6) linkage of residue **N** to residue **G**.

**Figure 6:**
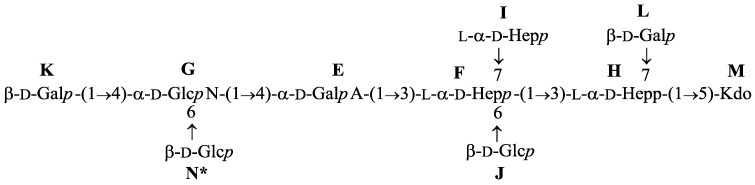
Structure of the core oligosaccharide from *P. shigelloides* O24 (strain CNCTC 92/89).

Comparison of the NMR data with the published data for the core oligosaccharide from the *P. shigelloides* O54:H2 (strain CNCTC 113/92) [2] showed that the core oligosaccharide is identical in the two strains of *P. shigelloides*. Thus, structural elucidation of OSI and OSII is not described in further details since the full characterization of an identical core (Figure 6) has been reported by Niedziela *et al.* [2].

## 3. Experimental Section

### 3.1. Bacteria

Bacteria *P. shigelloides* strain CNCTC 92/89 was classified as serotype O24:H8. This strain was obtained from the Institute of Hygiene and Epidemiology, Prague, Czech Republic. The bacteria were grown and harvested as described previously [15]. The LPS was extracted from bacterial cells of *P. shigelloides* O24 by the hot phenol/water method [16], dialyased, purified by ultracentrifugation and freeze-dried. The LPS was analyzed by SDS-PAGE according to the method of Laemmli [17] with modifications described previously [18] and visualized by the silver staining method [19].

### 3.2. Isolation of the O-Specific Polysaccharide

LPS of *P. shigelloides* O24 was isolated from the water phase by the hot phenol/water method with a yield of 1.7% and then analysed by SDS-PAGE. The LPS was degraded with 1.5% acetic acid containing 2% sodium dodecyl sulfate (SDS) at 100 °C for 15 min. The reaction mixture was freeze-dried and extracted five times with 96% ethanol to remove the SDS. The residue was then suspended in water and centrifuged. The supernatant (polysaccharide and oligosaccharide mixture) was lyophilized. The supernatant fraction was first fractioned by gel permeation chromatography (GPC) performed on a Superdex 75 (16/60) HiLoad column (GE Healthcare, Uppsala, Sweden) and then further fractioned on a Superdex 30 (16/60) HiLoad column (GE Healthcare), both equilibrated with 0.1 M ammonium acetate buffer at pH 7. The eluates were monitored with UV detector at 230 and 255 nm.

### 3.3. Electrospray Ionization Mass Spectrometry

ESI-MS was performed in both positive and negative mode using a Bruker maXis Impact Q-TOF mass spectrometer. The samples were dissolved in a 1:1 (v/v) water-methanol mixture and sprayed via direct injection at a flow rate of 2 μL/min.

### 3.4. NMR Spectroscopy

The PS, OSI and OSII fractions were repeatedly exchanged with D_2_O by lyophilisation before analysis. The NMR spectra of the fractions in D_2_O solutions were recorded with a Bruker AVANCE III 600 MHz spectrometer equipped with a 5 mm broadband observe detection SmartProbe and with a 5 mm ^1^H/^13^C/^15^N/^31^P QCP inverse detection Cryoprobe, both equipped with z-gradient. The ^13^C and ^1^H chemical shifts were measured using acetone as an internal standard (δ_H_ = 2.225 and δ_C_ 31.05). The data were acquired and processed using the Bruker software, TopSpin 3.1. The signals were assigned using 1D and 2D experiments, COSY, TOCSY, NOESY, HSQC with and without carbon decoupling, HSQC-TOCSY and HMBC, from the Bruker pulse sequence library. The TOCSY experiments were carried out with mixing times of 20, 90 and 120 ms, NOESY with 100, 300 and 500 ms, HSQC-TOCSY with 90 and 120 ms and HMBC with 10 ms.

## 4. Conclusions

So far, the structure of three complete LPS isolated from *P. shigelloides* CNCTC 113/92 (serotype O54) [2,20,21], CNCTC 144/92 (serotype O74) [22,23] and CNCTC 39/89 (serotype O37) [3] have been elucidated. Additionally, the core oligosaccharide substituted with the O-specific polysaccharide of serotypes O17 [24,25], O1 [26] and O33 strain CNCTC 34/89 [27], the O-specific polysaccharide of serotype O51 strain CNCTC 110/92 [28], strains 22074 and 12254 [29], and strain AM36565 [30] have been characterized. These studies have shown a few characteristics feature of *P. shigelloides* LPS’s, which is the lack of phosphate groups, the presence of uronic acid residues in the core oligosaccharide and the unusual hydrophobicity of the O-specific polysaccharides. It has been suggested that the presence of deoxy sugars with hydrophobic substituents could contribute to the overall hydrophobic nature of LPS’s. However, it has also been suggested that the main solubility factor might be conformational rather than compositional [31]. The possible role of the LPS-associated hydrophobicity has so far not been investigated.
